# Multimodal Feature-Level Fusion CBAM U-Net for Static Plantar Pressure Prediction Using Plantar Geometry and Sparse Anatomical Landmarks

**DOI:** 10.3390/s26134143

**Published:** 2026-07-01

**Authors:** Chongguang Wang, Kerrie Evans, Dean Hartley, Scott Morrison, Stuart McDonald, Martin Veidt, Gui Wang

**Affiliations:** 1 School of Mechanical and Mining Engineering, The University of Queensland, St Lucia, QLD 4072, Australiam.veidt@uq.edu.au (M.V.); 2 Healthia Limited, Bowen Hills, QLD 4006, Australia; 3 Faculty of Medicine and Health, The University of Sydney, Camperdown, NSW 2050, Australia; 4 iOrthotics Pty Ltd., Windsor, QLD 4030, Australia; 5 Institute of Rehabilitation Medicine and Health Care, Henan Academy of Innovations in Medical Science, Zhengzhou 451162, China; 6 Nantong Institute of Advanced Technology, Shanghai Jiaotong University, Nantong 226300, China

**Keywords:** plantar pressure prediction, multimodal fusion, CBAM U-Net, sparse-to-dense reconstruction, foot plantar geometry

## Abstract

Accurate plantar pressure distribution is important for biomechanics, gait analysis, rehabilitation, and diabetic foot assessment. However, wearable plantar pressure systems are often limited by sparse sensor layouts due to hardware complexity, power consumption, and user comfort constraints. This study proposes a multimodal deep learning framework for static plantar pressure prediction using plantar geometry information and sparse landmark constraints. A convolutional block attention module U-Net architecture was developed to integrate plantar geometry and sparse landmark modalities through dual-encoder feature fusion with attention refinement. Different network architectures, fusion strategies, and landmark densities were systematically evaluated using a controlled-variable experimental design. Results demonstrated that feature-level fusion consistently outperformed data-level fusion and unimodal configurations across all landmark densities. The proposed model achieved the best performance with a normalized root mean square error of 0.087 using 16 landmarks, and the same model maintained a normalized root mean square error of 0.138 using only two landmarks, indicating promising reconstruction performance even under highly sparse sensing conditions. Marginal contribution and synergy analyses further showed that feature-level fusion more effectively captured complementary interactions between plantar geometry and sparse anatomical guidance, particularly under sparse landmark conditions. These findings suggest that multimodal feature-level fusion provides an effective strategy for sparse-to-dense plantar pressure reconstruction and may support the development of low-cost intelligent insole systems for biomechanical monitoring and clinical applications.

## 1. Introduction

Plantar pressure distribution provides important biomechanical information regarding load transfer, postural stability, gait mechanics, and foot function during standing and locomotion [1,2]. Atypical plantar loading patterns are strongly associated with musculoskeletal disorders, diabetic foot ulceration, foot deformities, and gait impairments [3,4]. Consequently, plantar pressure analysis has become widely used in biomechanics, rehabilitation, sports science, orthotic evaluation, and clinical diagnosis [5]. Accurate plantar pressure assessment is particularly important for identifying regions exposed to excessive stress, which may contribute to tissue damage and long-term pathological progression in high-risk populations such as individuals with diabetes [6].

Conventional plantar pressure measurement systems can generally be categorized into platform-based systems and wearable in-shoe systems [2]. Platform-based systems, such as pressure mats and force plates, provide high spatial resolution and strong measurement repeatability under laboratory conditions. However, these systems are limited to short-duration measurements and controlled environments, restricting their applicability for continuous daily monitoring [7]. In contrast, wearable in-shoe systems enable ambulatory monitoring in real-world conditions but are constrained by sensor number, power consumption, comfort, durability, and hardware cost [7]. As a result, wearable systems often employ sparse sensor layouts rather than dense pressure arrays, leading to reduced spatial resolution and incomplete plantar pressure representation [8].

To address this limitation, recent studies have investigated sparse-to-dense plantar pressure reconstruction methods, where sparse sensor measurements are used to estimate full plantar pressure distributions using computational models [9,10]. Traditional reconstruction approaches are commonly based on interpolation, statistical modelling, or compressed sensing techniques [11]. However, plantar pressure distributions exhibit highly nonlinear spatial patterns influenced by foot morphology, anatomical structure, and biomechanical loading conditions, limiting the capability of conventional reconstruction methods [8].

Recent advances in deep learning have demonstrated strong performance in image reconstruction, multimodal learning, and sparse-to-dense estimation tasks [12,13]. In plantar pressure analysis, deep neural networks have been applied to estimate plantar loading from sparse pressure measurements, inertial sensors, or gait-related features [14,15]. U-Net-based encoder–decoder architectures are particularly suitable for spatial reconstruction problems because they combine multi-scale feature extraction with skip connections that preserve local spatial information [16]. Furthermore, attention mechanisms such as the Convolutional Block Attention Module (CBAM) can improve feature representation by adaptively emphasizing informative spatial regions and feature channels [16,17].

In addition to sparse pressure sensing, foot morphology has been shown to correlate strongly with plantar pressure distribution [18,19]. Variations in foot structure, such as arch height and plantar geometry, influence load transfer patterns across the plantar surface [20]. Advances in three-dimensional scanning technologies have enabled accurate acquisition of plantar geometry, providing additional biomechanical information that may support plantar pressure estimation [21]. However, most existing studies on plantar pressure reconstruction primarily rely on sparse pressure measurements alone, using machine learning or deep learning models to estimate dense plantar pressure distributions without incorporating plantar morphology information [8,9,10]. Consequently, the integration of plantar geometry and sparse landmark constraints within a multimodal deep learning framework remains largely unexplored.

Multimodal fusion has become increasingly important in medical imaging and biomechanical analysis because different modalities often contain complementary information [22,23]. Among existing fusion strategies, feature-level fusion has demonstrated strong capability for learning higher-order cross-modal relationships while preserving modality-specific representations [24,25]. Nevertheless, the effectiveness of different fusion strategies for integrating plantar geometry and sparse plantar landmarks remains insufficiently explored.

Therefore, this study proposes a multimodal plantar pressure prediction framework based on feature-level fusion CBAM U-Net architecture. The framework integrates plantar geometry information and sparse landmark constraints for static plantar pressure reconstruction. Different fusion strategies, network architectures, and landmark densities are systematically evaluated to investigate their contribution to plantar pressure prediction performance. In addition, marginal contribution and synergy analyses are conducted to quantify the complementary interaction between plantar geometry and sparse landmark information. The proposed framework aims to support the development of low-cost intelligent insole systems capable of reconstructing dense plantar pressure distributions from sparse measurements.

## 2. Materials and Methods

### 2.1. Study Design and Experimental Workflow

This study was approved by the Human Research Ethics Committee of The University of Queensland (Ethics Approval No. 2022/HE000445). Participants provided written informed consent prior to participation. Thirty-five healthy adult participants without known foot-related pathologies were recruited using convenience sampling from the university community. Participants were eligible if they were able to stand and walk independently during data acquisition. Participants represented a diverse range of foot sizes. The study cohort consisted of 24 males and 11 females, with a mean age of 26.8 ± 4.6 years, body weight of 70.0 ± 14.1 kg, and height of 174.5 ± 7.7 cm. Foot size was 25.7 ± 1.4 cm for the left foot and 25.4 ± 1.4 cm for the right foot.

**Figure 1:**
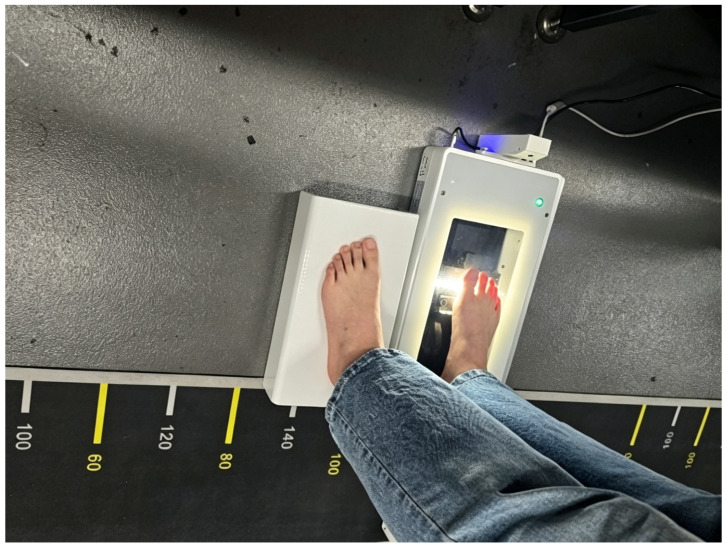
Experimental setup for acquiring plantar geometry using the USOL-X 3D laser foot scanner.

Foot plantar geometry data in stereolithography (STL) format were acquired using a USOL-X 3D Laser Foot Plantar Scanner (Vismach Technology Co., Ltd., Wuhan, China) with manufacturer-reported accuracy of 1.0 mm. Participants stood barefoot on the scanner and maintained a stable, upright posture during scanning to obtain three-dimensional models of the plantar surface (Figure 1). Each participant underwent one scan for both the left and right foot, resulting in a total of 70 plantar foot scan models after quality control procedures.

**Figure 2:**
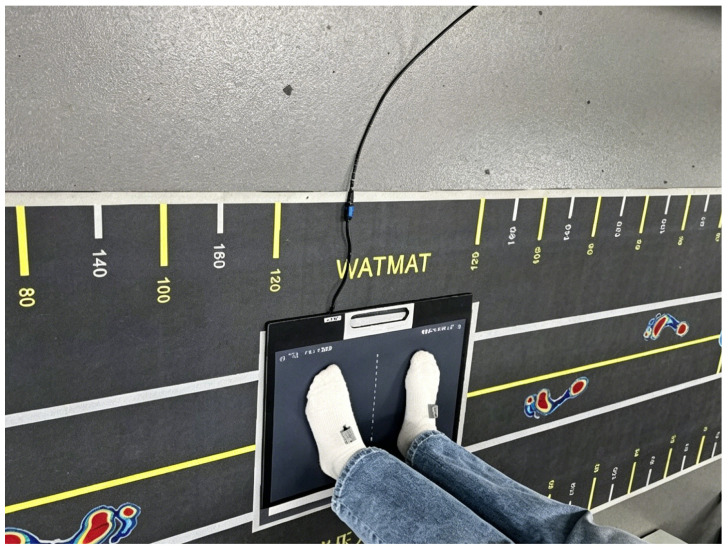
Experimental setup for static standing plantar pressure acquisition using the pressure plate system.

Static standing plantar pressure data were collected using a foot pressure plate WatMat 480 Plantar Pressure Measurement System (Vismach Technology Co., Ltd., Wuhan, China) with a spatial resolution of 64 × 96 sensors, with each sensor measuring 5 mm × 5 mm. This sensor density satisfies established requirements for capturing detailed plantar pressure distributions [2]. For each participant, static standing plantar pressure was recorded 10 times for both the left and right foot. During each trial, participants were instructed to remain still and evenly distribute their body weight to minimize measurement variability (Figure 2). After quality control, 692 plantar pressure distribution samples were retained for further analysis. Pairing involved matching each plantar pressure sample to the corresponding participant and plantar geometry image.

### 2.2. Multimodal Input Construction and Spatial Registration

To formulate plantar pressure prediction as an image-to-image translation problem, all inputs and outputs were standardized into aligned 64 × 64 image representations.

Raw plantar pressure measurements were separated into left-foot and right-foot regions and resized to 64 × 64 grayscale images. Zero-padding with a black background was applied to preserve plantar geometry during resizing. The pressure acquisition system generated 10-bit sensor intensity values (0–1023). Preliminary analysis of the dataset showed that the maximum recorded intensity was approximately 480. Therefore, a fixed normalization factor of 512 was selected to map pressure values into an 8-bit grayscale range [0, 255], ensuring consistent scaling across all samples while avoiding sample-dependent normalization:

(1) $$I_{scaled}(x,y)=\frac{256}{512}\cdot I(x,y)$$

where I(x,y) denotes the original sensor intensity value in the pressure frame.

Plantar geometry modalities were generated from the 3D scanning system. STL meshes were converted into dense point clouds using barycentric interpolation and projected onto a 64 × 64 image grid. Pixel intensities $J\left(x_{m},y_{n}\right)$ were generated from averaged and normalized height values:

(2) $$J\left(x_{m},\;y_{n}\right)=\frac{255}{N}\cdot \sum_{N}^{i=1}\frac{40-z_{i}}{40}$$

where the constant 40 mm represents the height range z used for grayscale normalization based on the scanner output range. The value of 40 mm was selected because it is near the maximum plantar height variation observed in the scanned dataset and therefore preserves most of anatomical shape range while avoiding excessive sensitivity to outliers. Values above this range were clipped before scaling and represent the projected height value in millimetres.

**Figure 3:**
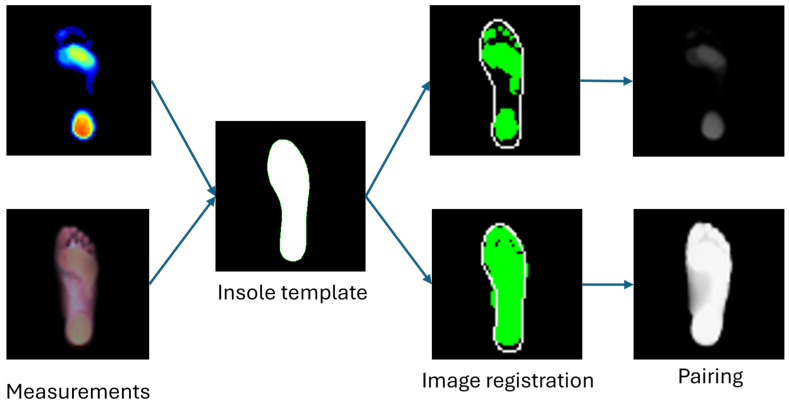
Spatial registration process used to align plantar geometry and pressure images to the size-specific insole template.

To ensure pixel-level correspondence between plantar geometry inputs and plantar pressure targets, all images were spatially registered to size-specific insole templates created for this study. These templates served as common registration targets and reference coordinate systems for landmark placement. Samples were first categorized into three foot-size groups: small (<24 cm), medium (24–26 cm), and large (>26 cm). Registration consisted of centroid alignment followed by iterative rigid transformation optimization. Registration quality was quantified using the overlap ratio between the plantar image mask and the corresponding insole template. During each iteration, rotation angles between 0° and 360° were exhaustively evaluated at 1° increments while keeping the translation fixed, and the angle producing the highest overlap ratio was selected. Subsequently, horizontal and vertical translations within a ±5 pixel range were exhaustively evaluated at 1-pixel increments while keeping the rotation fixed, and the translation producing the highest overlap ratio was selected. These rotation and translation optimization steps were repeated alternately until no further improvement in overlap ratio was observed. The final transformation with the highest overlap ratio was used for image registration (Figure 3).

**Figure 4:**
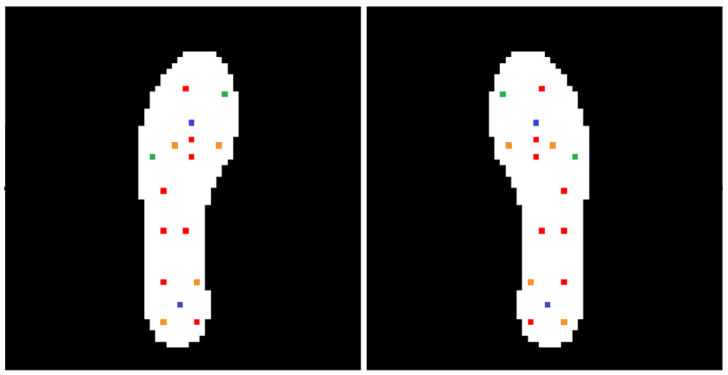
Anatomical 16-landmark configurations on the standardized insole template. Three reduced landmark subsets were derived to evaluate density effects: 2-landmark (heel, third metatarsal head; blue), 4-landmark (+fifth metatarsal head, hallux; green), and 8-landmark (+two additional heel points, first and fourth metatarsal heads; orange).

Sparse landmark maps were incorporated as an additional modality for plantar pressure prediction. Sixteen anatomical landmarks covering major load-bearing regions, including the heel, midfoot, metatarsal heads, and hallux, were selected based on previous sensor placement studies (Figure 4) [8,26,27].

After spatial registration, pressure values at the landmark coordinates were extracted and encoded into sparse 64 × 64 grayscale images, where only landmark locations contained non-zero intensities. To evaluate the effect of landmark density, reduced 2-, 4-, and 8-landmark configurations were derived from the full 16-landmark set. The reduced landmark configurations were selected using a principal component analysis (PCA)-based ranking approach. Pressure values from the full 16-landmark set were first extracted across all samples to construct a landmark feature matrix. PCA was then applied to this matrix, and each landmark was ranked according to its contribution to the variance captured by the principal components. Landmarks with higher variance contributions were considered more informative and were progressively retained to construct the 2-, 4-, and 8-landmark subsets. Landmark coordinates for each configuration are provided in Table 1.

### 2.3. Deep Learning Architectures

The plantar pressure reconstruction problem was formulated as a supervised image-to-image translation task:

(3) $$\hat{Y}=f(X;\theta )$$

where X represents the multimodal input, $\hat{Y}$ denotes the predicted plantar pressure distribution, and f represents the deep learning model parameterized by θ.

The input X consisted of one or more modalities depending on the experimental configuration, including: plantar geometry information and sparse landmark maps. The output corresponded to the aligned 64 × 64 grayscale plantar pressure image converted from measurement.

**Figure 5:**
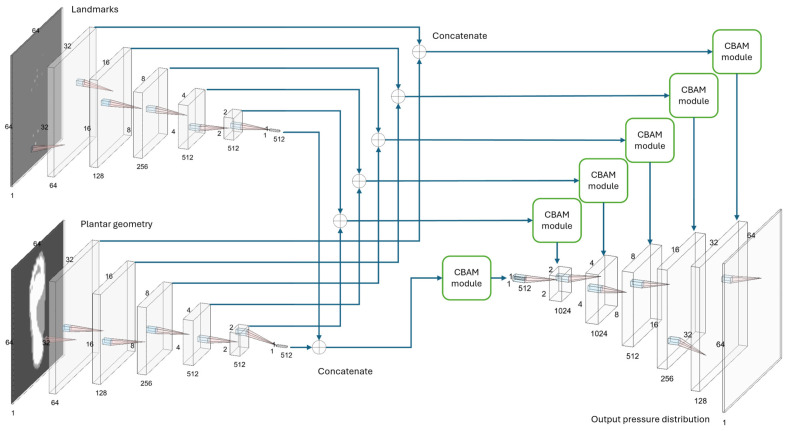
Proposed feature-level fusion CBAM U-Net architecture for multimodal plantar pressure prediction using plantar geometry and sparse landmark inputs.

The proposed feature-level fusion CBAM U-Net integrated plantar geometry information and sparse landmark constraints for plantar pressure prediction (Figure 5). The network employed a dual-encoder architecture, where plantar geometry and landmark modalities were processed separately before multi-scale feature fusion. Feature maps from corresponding encoder levels were concatenated and refined using CBAM attention modules. The fused features were further combined at the bottleneck layer, and the decoder reconstructed the 64 × 64 plantar pressure image using the fused multi-scale representations.

In addition to the proposed feature-level fusion CBAM U-Net, several U-Net variants were implemented to evaluate the effects of fusion strategy, landmark number, and input modality on plantar pressure prediction performance. To ensure fair comparison and controlled factor importance evaluation, all other U-Net variants were configured with identical parameters, including network depth, padding strategy, preprocessing, optimizer, loss functions, batch size, epoch number, and five-fold cross-validation strategy; only the target experimental factor was modified in each comparison. These variants included:

1. CBAM U-Net with data-level fusion, where plantar geometry images and sparse landmark maps were concatenated as multi-channel inputs and processed using a single shared encoder. Unlike feature-level fusion, the two modalities were merged directly at the input stage.
2. Classical U-Net models with feature-level fusion and data-level fusion were implemented as controlled comparison models to evaluate the influence of the CBAM attention mechanism.
3. Classical U-Net models with shape-only or landmark-only inputs were implemented as baseline models to evaluate the independent contribution of each modality. These baseline models retained the original U-Net skip connection structure without CBAM attention modules.

Different combinations of network architectures, input configurations, fusion strategies, and landmark densities were systematically evaluated. Classical U-Net and CBAM-enhanced U-Net architectures were implemented to assess the effect of attention mechanisms. Four input configurations, including shape-only, landmark-only, data-level fusion, and feature-level fusion, were investigated together with 2-, 4-, 8-, and 16-landmark configurations to analyse the influence of sparse spatial guidance and multimodal fusion on plantar pressure prediction performance.

### 2.4. Training Configuration

To ensure fair comparison across all experiments, identical preprocessing, optimization, and training settings were applied.

A five-fold cross-validation strategy was adopted. All samples from a participant were assigned to the same fold to prevent subject leakage between training and validation sets. The folds were stratified according to foot size and sex distributions.

The total loss function consisted of three components:

(4) $$L_{total}=L_{MAE}+\lambda_{1}L_{sum}+\lambda_{2}L_{TV}$$

where: $L_{MAE}$ denotes mean absolute error; $L_{sum}$ constrains global pressure magnitude consistency; $L_{TV}$ represents total variation loss. The Adam optimizer was used with the following hyperparameters: initial learning rate: 2 × $10^{-4}$; betas: (0.5, 0.999); weight decay: 1 × $10^{-5}$. The learning rate was reduced by a factor of 0.5 every 20 epochs.

Training was performed for 51 epochs with a batch size of 64. Gradient clipping with a maximum norm of 1.0 was applied. Mixed-precision training using automatic mixed precision and gradient scaling was implemented to improve computational efficiency. All experiments were conducted using Python 3.12 (Anaconda Distribution 2024.10, Anaconda Inc., Austin, TX, USA), with PyTorch (Version 2.3.0, Meta Platforms Inc., Menlo Park, CA, USA) built against the NVIDIA CUDA Toolkit (Version 12.1, NVIDIA Corp., Santa Clara, CA, USA) acceleration on an NVIDIA RTX 3070 Ti GPU.

### 2.5. Evaluation Index

Performance was evaluated using average ranged-normalized root mean square error (NRMSE) and confidence intervals (CI). NRMSE was used as the main comparison metric to reflect model stability and generalization consistency across folds and repeated measurements.

For each prediction–ground truth pair (predicted plantar pressure map $\hat{Y}$ and ground truth Y), NRMSE is computed only over the non-zero plantar-contact region defined by the force-plate reference. Let $Ω=\left\{\left.i\right|Y_{i}>0\right\}$ be the index set of non-zero pixels in the ground-truth map and let N=∣Ω∣. Then, NRMSE is defined as:

(5) $$NRMSE=\frac{\sqrt{\frac{1}{N}\sum_{i=1}^{N}({\hat{Y}}_{i}-Y_{i}{)}^{2}}}{255}$$

The divisor 255 was used because all input and target images were normalized to an 8-bit grayscale range [0, 255] during preprocessing. After NRMSE is computed for each pair, the final mean NRMSE score is obtained by taking the average across all pairs. In the context of image reconstruction and regression tasks, lower NRMSE values indicate better fidelity to the reference image.

To quantify the effect of multimodal fusion, marginal contribution, gain, and synergy were evaluated for the U-Net variants. These metrics were used to assess the individual contributions of plantar geometry and landmark information, as well as their complementary interaction in improving prediction performance.

Letting ${NRMSE}_{S}$ denote the error of the shape-only model, ${NRMSE}_{L}$ the error of the landmark-only model, and ${NRMSE}_{F}$ the error of a fusion model, the marginal contribution of fusion relative to a single modality is defined as the improvement obtained by adding the other modality:

(6) $$\left\{\begin{array}{c} \Delta_{S}={NRMSE}_{S}-{NRMSE}_{F}\\ \Delta_{L}={NRMSE}_{L}-{NRMSE}_{F}\; \end{array}\right.$$

where $\Delta_{S}$ represents the error reduction when landmark information is added to the shape baseline, and $\Delta_{L}$ represents the error reduction when shape information is added to the landmark baseline. These values directly quantify how much fusion improves upon each single-modality model and serve as evidence of each modality’s practical contribution.

To evaluate whether fusion truly provides new information beyond the stronger single modality, a baseline error ${NRMSE}_{0}$ is defined as the worst single-modality performance:

(7) $${{NRMSE}_{0}}^{\left(i\right)}=max({{NRMSE}_{S}}^{\left(i\right)},{{NRMSE}_{L}}^{\left(i\right)})$$

The gain from baseline is then defined as:

(8) $$\bar{{Gain}_{X}}=\frac{1}{N}\sum_{i=1}^{N}\left({{NRMSE}_{0}}^{\left(i\right)}-{{NRMSE}_{X}}^{\left(i\right)}\right),\;X\in \left(S,L,F\right)$$

A positive gain indicates that fusion outperforms the best single-modality model; if gain is near zero, the fusion model behaves similarly to the dominant modality.

To further interpret whether fusion provides complementary information (rather than simply inheriting one modality), synergy was defined as the amount of improvement beyond what would be expected under single-modality dominance. Since dominance is represented by ${NRMSE}_{0}$, synergy is equivalent to gain in this framework:

(9) $$\mathrm{Synergy}=\frac{1}{N}\sum_{i=1}^{N}\left({Gain}_{F}-{Gain}_{S}-{Gain}_{L}\;\right)$$

Under this definition, synergy > 0 implies that shape and landmark modalities provide non-redundant and complementary cues that improve prediction quality when combined. Conversely, synergy ≤ 0 suggests that the additional modality does not contribute meaningful information beyond the dominant one or introduces noise, making fusion unnecessary under the tested conditions [28].

## 3. Results

**Figure 6:**
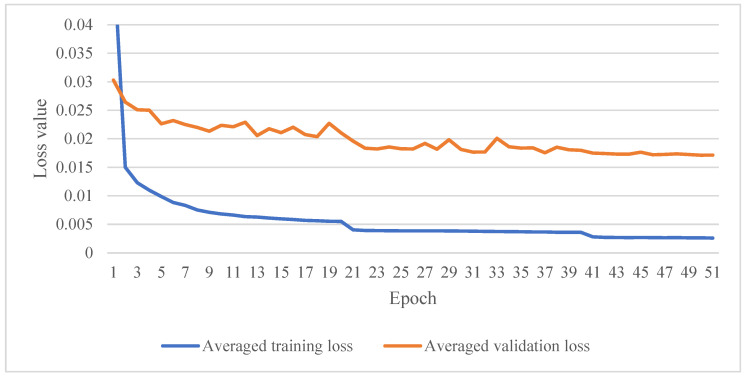
Averaged training and validation loss curves of the proposed CBAM-enhanced feature-level fusion U-Net with 16 landmarks over 51 training epochs. Loss values represent the mean across five cross-validation folds.

Figure 6 presents the training and validation loss curves of the proposed CBAM-enhanced feature-level fusion U-Net with 16 landmarks. The reported losses represent the average values across the five cross-validation folds. Both losses gradually stabilized after approximately 40 epochs, indicating stable convergence and limited evidence of overfitting. and suggesting that the model had effectively converged under the selected training configuration.

**Figure 7:**
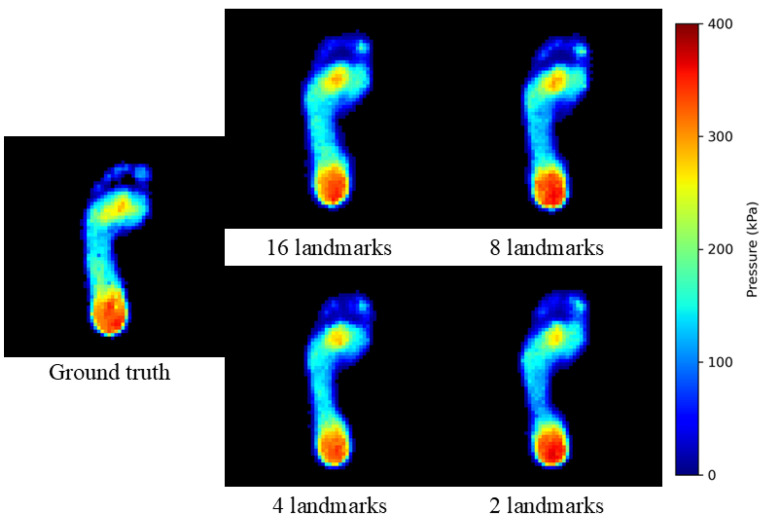
Representative plantar pressure reconstruction results of the proposed CBAM-enhanced feature-level fusion model under different landmark densities. The measured ground-truth plantar pressure distribution is compared with the corresponding predicted distributions generated using 2-, 4-, 8-, and 16-landmark configurations. All pressure maps are displayed using the same false-colour scale.

Figure 7 presents representative plantar pressure reconstruction results generated by the proposed CBAM-enhanced feature-level fusion model under 2-, 4-, 8-, and 16-landmark configurations. The predicted pressure maps successfully captured the major plantar loading regions observed in the ground-truth distribution, including the heel and forefoot regions, across all landmark densities. Although reconstruction errors were more apparent under the 2-landmark condition, the overall pressure distribution pattern remained consistent with the ground truth. Increasing landmark density progressively improved the recovery of local pressure details and high-pressure regions, with the 16-landmark configuration exhibiting the closest visual agreement with the measured plantar pressure distribution.

### 3.1. Prediction Performance Based on NRMSE

The prediction performance of different network architectures, input configurations, fusion strategies, and landmark densities was evaluated using mean NRMSE. The results are summarized in Table 2, including NRMSE within the foot area, 95% CI within the foot area, and NRMSE over the full image.

Overall, the results demonstrated that both network architecture and input configuration significantly influenced plantar pressure prediction performance. Among all evaluated configurations, the CBAM-enhanced U-Net with feature-level fusion and 16 landmark constraints achieved the best overall performance, with an NRMSE of 0.087 ± 0.003 within the foot area. The classical U-Net with feature-level fusion and 16 landmarks achieved comparable performance, with an NRMSE of 0.089 ± 0.003.

#### 3.1.1. Effect of Fusion Strategy

**Figure 8:**
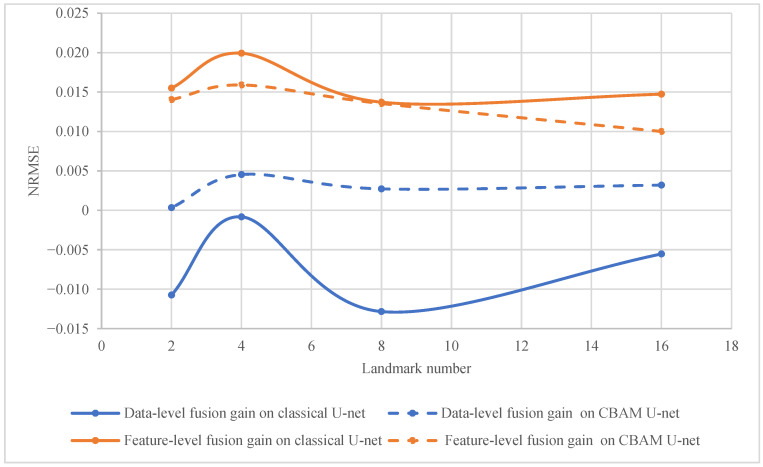
Gain analysis of data-level fusion and feature-level fusion configurations for classical U-Net and CBAM-enhanced U-Net models across different landmark densities. Markers indicate the evaluated landmark configurations. Gain values were calculated relative to the corresponding landmark-only baseline of each network architecture.

Feature-level fusion consistently outperforms data-level fusion and landmark-only configurations across all landmark densities for both network architectures. In the CBAM-enhanced U-Net, feature-level fusion reduced the NRMSE from 0.094 to 0.087 at the 16-landmark configuration compared with data-level fusion, with similar improvements observed for other landmark densities. Gain analysis (Figure 8) further showed that feature-level fusion consistently achieved positive gain values across all landmark configurations, indicating improved prediction performance relative to landmark-only models. In contrast, data-level fusion produced limited or negative gain, particularly in the classical U-Net, suggesting that latent feature fusion was more effective than direct input concatenation for multimodal integration.

#### 3.1.2. Effect of Landmark Number

The influence of landmark density was also clearly observed. For both network architectures and all fusion strategies, increasing the number of landmarks consistently improved prediction performance.

**Figure 9:**
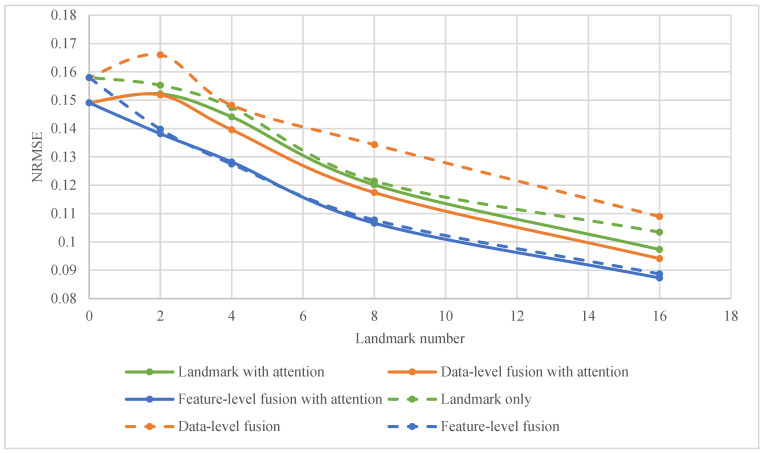
Comparison of NRMSE performance trends for different network architectures and fusion strategies as landmark density increases from 0 to 16 landmarks.

Figure 9 illustrates the influence of landmark density on plantar pressure prediction performance across different network architectures and fusion strategies. The NRMSE values decreased progressively as the landmark number increased from 0 to 16, indicating that higher landmark density provided stronger spatial constraints for plantar pressure reconstruction. Among all evaluated configurations, feature-level fusion achieved the lowest NRMSE values across both classical U-Net and CBAM-enhanced U-Net architectures. The feature-level fusion with attention configuration achieved the best overall performance at 16 landmarks. The results also demonstrated that CBAM attention mechanisms improved prediction performance for both landmark-only and data-level fusion configurations, particularly at lower landmark densities.

#### 3.1.3. Effect of Attention Mechanism

**Figure 10:**
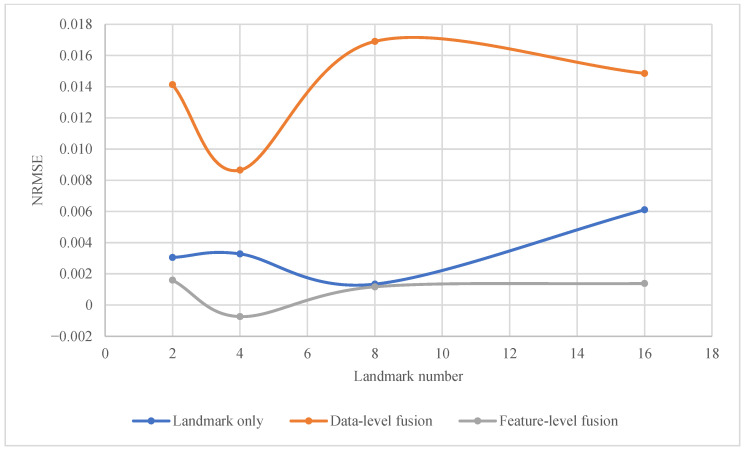
Relative performance gain of the CBAM-enhanced U-Net compared with the classical U-Net across different landmark densities.

Comparing the two network architectures, the CBAM-enhanced U-Net generally achieved slightly lower NRMSE values than the classical U-Net under most experimental conditions, particularly in data-level fusion and landmark-only configurations. However, the performance difference between the two architectures became smaller under feature-level fusion (Figure 10). This observation indicates that attention mechanisms contributed additional benefits for spatial feature extraction, although the fusion strategy and landmark density had a greater overall influence on prediction performance.

### 3.2. Marginal Contribution and Synergy

To further investigate the contribution of foot plantar geometry and plantar landmarks in plantar pressure prediction, the marginal contribution and synergy metrics were evaluated for both data-level fusion and feature-level fusion under different numbers of landmarks (Table 3). The reported values were averaged across the entire dataset. The baseline error was defined as the weaker single-modality performance for each sample, and the gain and synergy metrics quantified the improvement achieved through multimodal fusion.

Marginal contribution analysis showed that landmark information contributed more strongly to plantar pressure prediction than foot plantar geometry across all configurations. In feature-level fusion, shape contribution remained consistently positive, ranging from 0.010 to 0.016, whereas data-level fusion showed minimal contribution from shape information, particularly at the 2-landmark configuration. Landmark contribution also remained consistently higher in feature-level fusion, achieving values of 0.062, 0.042, 0.021, and 0.011 for the 16-, 8-, 4-, and 2-landmark configurations, respectively. In contrast, data-level fusion showed reduced landmark contribution as landmark density decreased and became negative at the 2-landmark configuration (−0.003). These results indicate that feature-level fusion is more effectively preserved and integrated both global plantar geometry and sparse anatomical guidance, particularly under sparse landmark conditions.

Feature-level fusion consistently demonstrated positive synergy across all landmark configurations, with synergy values of 0.010, 0.012, 0.009, and 0.004 for the 16-, 8-, 4-, and 2-landmark configurations, respectively. The highest synergy was observed at the 8-landmark configuration, indicating the strongest complementary interaction between plantar geometry and landmark information. In contrast, data-level fusion produced only marginal synergy at high landmark densities and negative synergy at the 4- and 2-landmark configurations. Similarly, feature-level fusion achieved consistently larger fusion gains than data-level fusion across all landmark configurations. These findings suggest that feature-level fusion captured complementary multimodal relationships more effectively than direct input concatenation.

## 4. Discussion

This study investigated the use of multimodal deep learning for static plantar pressure prediction by integrating plantar geometry information and sparse landmark constraints using U-Net-based architectures. The results demonstrated that feature-level fusion consistently outperformed data-level fusion and unimodal configurations across all landmark densities, indicating that latent multimodal integration was more effective than direct input concatenation for plantar pressure reconstruction.

The superiority of feature-level fusion suggests that plantar geometry and sparse landmark information provide complementary biomechanical information at different spatial scales. Plantar geometry mainly contributed global structural information related to foot morphology, while sparse landmarks provided localized pressure constraints at anatomically important regions. By processing these modalities independently before fusion, the proposed dual-encoder architecture preserved modality-specific features and enabled more effective multimodal interaction through CBAM attention refinement. In contrast, data-level fusion showed limited or negative gain under sparse landmark conditions, suggesting that early input concatenation may dilute sparse landmark information within dense image representations.

The marginal contribution analysis further demonstrated that landmark information contributed more strongly to prediction performance than plantar geometry alone. This observation is biomechanically reasonable because landmark constraints directly encode localized pressure values associated with major load-bearing regions. However, plantar geometry still provided consistent positive contributions in feature-level fusion, particularly when landmark density was low. This indicates that foot morphology contained complementary information capable of compensating for sparse spatial guidance. The positive synergy values observed in feature-level fusion further supports the existence of cooperative interaction between global foot structure and local anatomical constraints.

Negative synergy observed in data-level fusion under sparse landmark conditions suggests that direct input concatenation may not effectively preserve sparse landmark information. When only a few landmark pixels are present, the sparse signal can be overwhelmed by the much denser plantar geometry modality, causing the network to rely primarily on shape information. Consequently, the additional landmark modality may contribute little useful information and can even introduce noise, resulting in negative synergy values. Feature-level fusion mitigates this problem by preserving modality-specific representations before fusion [28].

Another important observation was that prediction performance gradually improved with increasing landmark density. Higher landmark numbers provided denser spatial guidance and reduced reconstruction ambiguity, particularly in high-pressure regions such as the heel and metatarsal heads. Nevertheless, feature-level fusion maintained stable performance gains even at low landmark densities, suggesting potential applicability for low-cost sparse-sensor wearable systems where minimizing sensor count is important for comfort, power consumption, and hardware complexity.

Compared with previous sparse plantar pressure reconstruction studies that relied primarily on pressure-only sensing [9,10,14,15], the proposed framework incorporated additional plantar geometry information to improve reconstruction robustness. The results suggest that combining morphology-related information with sparse plantar sensing may provide a feasible strategy for intelligent insole systems and personalized plantar pressure estimation.

Several limitations should be acknowledged. First, the study only included static standing plantar pressure data collected from healthy participants; therefore, the generalizability to dynamic gait conditions and pathological populations remains unclear. Although the proposed framework may support future applications in diabetic foot monitoring, orthotic evaluation, rehabilitation assessment, and other wearable plantar pressure monitoring scenarios, its clinical utility remains hypothetical until validated in patient populations. Several barriers to clinical deployment also remain, including the need for validation in pathological cohorts, robustness under real-world gait conditions, inter-subject variability, and integration with wearable sensing systems suitable for long-term monitoring.

Second, although the proposed framework demonstrated strong reconstruction performance, the dataset size was relatively limited compared with large-scale deep learning datasets. Repeated measurements increased the dataset size and captured natural standing variability, subject-wise cross-validation was adopted to minimize potential bias arising from intra-subject correlation and repeated observations from the same participant. Third, the sparse landmark subsets were selected according to anatomical relevance and prior sensor-placement studies rather than through an exhaustive optimization of all possible landmark combinations. Therefore, the current 2-, 4-, and 8-landmark configurations may not represent the globally optimal sparse layouts, and different landmark locations may further improve low-sensor reconstruction performance.

Future research will address these limitations by extending the framework to dynamic gait conditions, pathological populations, and larger datasets. Particular emphasis should be placed on evaluating the proposed framework in clinical populations, including individuals with diabetic foot disease, foot deformities, and gait impairments, to establish its clinical validity and practical utility. Additional multimodal inputs, such as IMU signals and temporal gait features, may further improve plantar pressure estimation and support real-time wearable monitoring applications [29]. Future studies should also evaluate alternative sparse landmark configurations or data-driven sensor-placement optimization to identify the most informative low-sensor layouts for wearable deployment. In addition, the proposed multimodal fusion framework should be evaluated using other state-of-the-art architectures, such as ResUNet, UNet++, and Attention U-Net [13], to further investigate the interaction between network architecture, fusion strategy, and modality contribution in plantar pressure reconstruction.

## 5. Conclusions

This study proposed a multimodal deep learning framework for static plantar pressure prediction using plantar geometry and sparse landmark constraints. The proposed feature-level fusion CBAM U-Net consistently outperformed data-level fusion and unimodal configurations across all landmark densities, achieving the best performance with an NRMSE of 0.087 using 16 landmarks and maintaining an NRMSE of 0.138 using only two landmarks. Marginal contribution and synergy analyses further demonstrated that feature-level fusion more effectively captured complementary interactions between plantar geometry and sparse anatomical guidance. The findings suggest that multimodal feature-level fusion provides an effective strategy for sparse-to-dense plantar pressure reconstruction and may support the development of low-cost intelligent plantar pressure monitoring systems.

## Figures and Tables

**Table 1:** Coordinates of the 16 anatomical landmark locations for small, medium, and large right-foot templates in the standardized 64 × 64 image space. Coordinates were defined in a 64 × 64 image space with the origin located at the top-left corner, x increasing from left to right and y increasing from top to bottom. Left-foot coordinates were generated by horizontal mirroring. Blue, green, and orange labels indicate landmarks included in the 2-, 4-, and 8-landmark configurations, respectively, and these colours are also indicated in Figure 4.

	Small (<24 cm)		Medium (24~26 cm)		Large (>26 cm)	
ID	x	y	x	y	x	y
1	31	15	31	14	31	12
2	30	21	30	20	30	19
3	33	25	33	24	33	24
4 (green)	37	26	37	26	38	25
5 (orange)	35	32	35	32	36	32
6	35	38	35	39	36	39
7	35	47	35	48	36	50
8 (orange)	35	53	35	55	36	57
9 (green)	25	17	24	16	24	14
10 (orange)	26	25	26	24	25	24
11 (blue)	30	24	30	23	30	23
12	30	26	30	26	30	25
13	31	38	31	39	31	39
14 (orange)	29	47	29	48	28	50
15 (blue)	32	50	32	52	32	53
16	29	53	29	55	28	57

**Table 2:** Quantitative comparison of plantar pressure prediction performance for different network architectures, fusion strategies, and landmark configurations based on NRMSE.

Model	Fusion/Input	Landmarks	NRMSE Foot Area	95% CI Foot Area	NRMSE Full Image
CBAM attention	Data-level	16	0.094	±0.004	0.035
CBAM attention	Feature-level	16	0.087	±0.003	0.032
CBAM attention	Landmark	16	0.097	±0.004	0.036
CBAM attention	Data-level	8	0.117	±0.004	0.044
CBAM attention	Feature-level	8	0.107	±0.005	0.040
CBAM attention	Landmark	8	0.120	±0.005	0.045
CBAM attention	Data-level	4	0.140	±0.006	0.052
CBAM attention	Feature-level	4	0.128	±0.005	0.048
CBAM attention	Landmark	4	0.144	±0.006	0.055
CBAM attention	Data-level	2	0.152	±0.006	0.057
CBAM attention	Feature-level	2	0.138	±0.007	0.051
CBAM attention	Landmark	2	0.154	±0.006	0.058
CBAM attention	Shape	0	0.149	±0.006	0.056
Classical U-net	Data-level	16	0.109	±0.006	0.041
Classical U-net	Feature-level	16	0.089	±0.003	0.033
Classical U-net	Landmark	16	0.103	±0.004	0.038
Classical U-net	Data-level	8	0.134	±0.009	0.051
Classical U-net	Feature-level	8	0.108	±0.005	0.041
Classical U-net	Landmark	8	0.121	±0.005	0.046
Classical U-net	Data-level	4	0.148	±0.007	0.056
Classical U-net	Feature-level	4	0.127	±0.006	0.049
Classical U-net	Landmark	4	0.147	±0.006	0.055
Classical U-net	Data-level	2	0.166	±0.008	0.062
Classical U-net	Feature-level	2	0.140	±0.007	0.052
Classical U-net	Landmark	2	0.155	±0.007	0.059
Classical U-net	Shape	0	0.159	±0.007	0.061

**Table 3:** Dataset-averaged marginal contribution, fusion gain, and synergy of foot plantar geometry and landmark information under data-level and feature-level fusion with different numbers of landmarks. Baseline error represents the weaker single-modality baseline.

Fusion	Landmarks	NRMSE Foot Area	Shape Marginal Contribution	Landmark Marginal Contribution	Baseline Error	Fusion Gain	Synergy
Data-level	16	0.094	0.003	0.055	0.150	0.055	0.002
Feature-level	16	0.087	0.010	0.062	0.150	0.062	0.010
Data-level	8	0.117	0.003	0.032	0.151	0.034	0.001
Feature-level	8	0.107	0.014	0.042	0.151	0.044	0.012
Data-level	4	0.140	0.005	0.010	0.156	0.017	−0.003
Feature-level	4	0.128	0.016	0.021	0.156	0.028	0.009
Data-level	2	0.152	0.000	−0.003	0.159	0.008	−0.001
Feature-level	2	0.138	0.014	0.011	0.159	0.021	0.004

## Data Availability

The datasets generated and analysed during the current study are not publicly available due to participant privacy and ethics restrictions but are available from the corresponding author upon reasonable request. The deep learning training and preprocessing scripts used in this study are publicly available at: https://github.com/Wonder741/CBAM-U-Net-Feature-Fusion-Plantar-Pressure-Prediction accessed on 1 June 2026.

## References

[1] Arzehgar A., Nia R.G.N.N., Hoseinkhani M., Masoumi F., Sayyed-Hosseinian S.-H., Eslami S. (2025). An overview of plantar pressure distribution measurements and its applications in health and medicine. Gait Posture.

[2] Chen J.L., Dai Y.N., Grimaldi N.S., Lin J.J., Hu B.Y., Wu Y.F., Gao S. (2022). Plantar pressure-based insole gait monitoring techniques for diseases monitoring and analysis: A review. Adv. Mater. Technol..

[3] Castro-Martins P., Marques A., Coelho L., Vaz M., Baptista J.S. (2024). In-shoe plantar pressure measurement technologies for the diabetic foot: A systematic review. Heliyon.

[4] Jiang X., Li J., Zhu Z., Liu X., Yuan Y., Chou C., Yan S., Dai C., Jia F. (2024). Moveport: Multimodal dataset of EMG, IMU, motion capture, and insole pressure for analyzing abnormal movements and postures in rehabilitation training. IEEE Trans. Neural Syst. Rehabil. Eng..

[5] Amaro C.M., Paulino M.F., Valvez S., Roseiro L., Castro M.A., Amaro A.M. (2025). Comparative analysis of pressure platform and insole devices for plantar pressure assessment. Appl. Sci..

[6] Ren Y., Wang H., Song X., Wu Y., Lyu Y., Zeng W. (2024). Advancements in diabetic foot insoles: A comprehensive review of design, manufacturing, and performance evaluation. Front. Bioeng. Biotechnol..

[7] Santos V.M., Gomes B.B., Neto M.A., Amaro A.M. (2024). A systematic review of insole sensor technology: Recent studies and future directions. Appl. Sci..

[8] Fuchs P.X., Chen W.H., Shiang T.Y. (2024). Center of pressure measurement accuracy via insoles with a reduced pressure sensor number during gaits. Sensors.

[9] Chan H.L., Liang J.R., Chang Y.J., Chen R.S., Kuo C.C., Hsu W.Y., Tsai M.T. (2025). Enhancing plantar pressure distribution reconstruction with conditional generative adversarial networks from multi-region foot pressure sensing. Biomed. Signal Process. Control..

[10] Mun F., Choi A. (2022). Deep learning approach to estimate foot pressure distribution in walking with application for a cost-effective insole system. J. Neuroeng. Rehabil..

[11] Konovalov A.B. (2024). Compressed-sensing-inspired reconstruction algorithms in low-dose computed tomography: A review. Phys. Medica.

[12] Oscanoa J.A., Middione M.J., Alkan C., Yurt M., Loecher M., Vasanawala S.S., Ennis D.B. (2023). Deep learning-based reconstruction for cardiac MRI: A review. Bioengineering.

[13] Azad R., Aghdam E.K., Rauland A., Jia Y., Avval A.H., Bozorgpour A., Karimijafarbigloo S., Cohen J.P., Adeli E., Merhof D. (2024). Medical image segmentation review: The success of u-net. IEEE Trans. Pattern Anal. Mach. Intell..

[14] Moon J., Lee D., Jung H., Choi A., Mun J.H. (2022). Machine learning strategies for low-cost insole-based prediction of center of gravity during gait in healthy males. Sensors.

[15] Ren J.L., Wang A.H., Li H.Y., Yue X.B., Meng L. (2023). A transformer-based neural network for gait prediction in lower limb exoskeleton robots using plantar force. Sensors.

[16] Jumat J., Shapri A.H.M., Rahman N.A., Zakaria S.M.M.S., Kamarudin L.M. (2024). A systematic review of attention mechanisms in U-Net models for medical image segmentation. Proceedings of the International Conference on Electronic Design.

[17] Aboudi F., Drissi C., Kraiem T. (2024). A hybrid model for Ischemic stroke brain segmentation from MRI images using CBAM and ResNet50-UNet. Int. J. Adv. Comput. Sci. Appl..

[18] Gwani A.S., Asari M.A., Ismail Z.M. (2017). How the three arches of the foot intercorrelate. Folia Morphol..

[19] Jandova S., Mendricky R. (2022). Benefits of 3D printed and customized anatomical footwear insoles for plantar pressure distribution. 3D Print. Addit. Manuf..

[20] Weidensager L., Krumm D., Potts D., Odenwald S. (2024). Estimating vertical ground reaction forces from plantar pressure using interpretable high-dimensional approximation. Sports Eng..

[21] Zhou L., Wu G., Zuo Y., Chen X., Hu H. (2024). A comprehensive review of vision-based 3D reconstruction methods. Sensors.

[22] Zhao F., Zhang C., Geng B. (2024). Deep multimodal data fusion. ACM Comput. Surv..

[23] Li Y., Daho M.E.H., Conze P.-H., Zeghlache R., Le Boité H., Tadayoni R., Cochener B., Lamard M., Quellec G. (2024). A review of deep learning-based information fusion techniques for multimodal medical image classification. Comput. Biol. Med..

[24] Tang Q., Liang J., Zhu F. (2023). A comparative review on multi-modal sensors fusion based on deep learning. Signal Process..

[25] Naseem M.T., Seo H., Kim N.-H., Lee C.-S. (2024). Pathological gait classification using early and late fusion of foot pressure and skeleton data. Appl. Sci..

[26] Zhong F.Y., He L., Zavatsky A., Maiolino P. (2023). A sensor placement benchmarking method with principal component analysis. IEEE Trans. Instrum. Meas..

[27] Cleland L.D., Rowland H.M., Mazzà C., Saal H.P. (2023). Complexity of spatio-temporal plantar pressure patterns during everyday behaviours. J. R. Soc. Interface.

[28] Lin T., Ren Z., Zhu L., Zhu Y., Feng K., Ding W., Yan K., Beer M. (2025). A systematic review of multi-sensor information fusion for equipment fault diagnosis. IEEE Trans. Instrum. Meas..

[29] Qian Y., Sun D., Xia Z., Shao E., Song Y., Sárosi J., Bíró I., Gao Z., Gu Y. (2025). Estimating dynamic plantar pressure distribution from wearable inertial sensors using a hybrid CNN-BiLSTM architecture. Acta Bioeng. Biomech..

